# Pacific Ocean decadal forcing of long-term changes in the western Pacific subtropical high

**DOI:** 10.1038/srep37765

**Published:** 2016-11-30

**Authors:** Shinji Matsumura, Takeshi Horinouchi

**Affiliations:** 1 Faculty of Environmental Earth Science, Hokkaido University, Kita 10 Nishi 5, Sapporo 060-0810, Japan

## Abstract

The western Pacific subtropical high (WPSH) has a significant effect on droughts, heat waves, and tropical cyclone tracks over East Asia and the northwest Pacific. The WPSH has intensified during the past three decades, but its causes are not yet well understood. Here we show that the Pacific Decadal Oscillation (PDO) is responsible for the long-term changes in the WPSH through the meridional shift of the subtropical jet, based on comprehensive data analysis and model results. El Niño–Southern Oscillation (ENSO) is the leading forcing of WPSH variability over interannual timescales, whereas the PDO accounts for its low-frequency variability, resulting in it being independent of ENSO with regard to WPSH variability. The PDO in summer can be interpreted as a coupling with the WPSH. Our results provide useful information for projecting long-term changes in the WPSH.

The East Asian summer monsoon (EASM) and the western Pacific subtropical high (WPSH) each has a significant effect on rainfall, heat waves, and tropical cyclone tracks over East Asia and the northwest (NW) Pacific^1^,^2^,^3^,^4^. For many decades the EASM has shown different changes between East Asia and the NW Pacific. Over East Asia the EASM circulation and precipitation have weakened^5^,^6^. Many studies have proposed possible mechanisms for the weakening of the EASM, but its causes remain elusive. In contrast, over the NW Pacific the mechanisms associated with changes in the EASM have become increasingly clear. Over the past three decades, the EASM rainband over the NW Pacific has shifted northwards, contributing to the weakening of the Okhotsk high and intensification of the WPSH via the response to the EASM condensation heating^7^. The EASM rainband is shifted northwards with the subtropical jet as a result of increased sea surface temperatures (SSTs) in the Kuroshio and Oyashio Extension (KOE) region^8^, where a strong northward-decreasing SST gradient develops.

The KOE SST variability is dominated by the Pacific Decadal Oscillation (PDO), which is regarded as an air–sea interaction in the North Pacific, especially between the Aleutian low and the KOE SST in winter and spring, when the Aleutian low develops^9^,^10^. In summer, however, as the North Pacific subtropical high or the WPSH develops to take the place of the Aleutian low, the PDO cannot be explained by the same mechanism that drives in winter. Although summer is the season when El Niño–Southern Oscillation (ENSO) begins to develop, over the northwest Pacific, the tropical Indian Ocean SST warming in the summer of ENSO decaying years leads to the intensification of the WPSH^11^,^12^,^13^. The interannual and decadal time scales of the PDO are related to ENSO, but there are also independent signals whose variability is concentrated mainly along the KOE^14^. Many previous studies of the PDO have focused on winter, and few studies have discussed summer. Although the summer PDO is suggested as one of the possible factors for the weakening of the EASM^15^, it remains unclear how the PDO plays a role in the East Asian and NW Pacific summer climate. In the present study, we provide robust evidence that the summer PDO plays a critical role in East Asian and NW Pacific summer climate change by enhancing WPSH variability.

## Results

**Figure 1:**
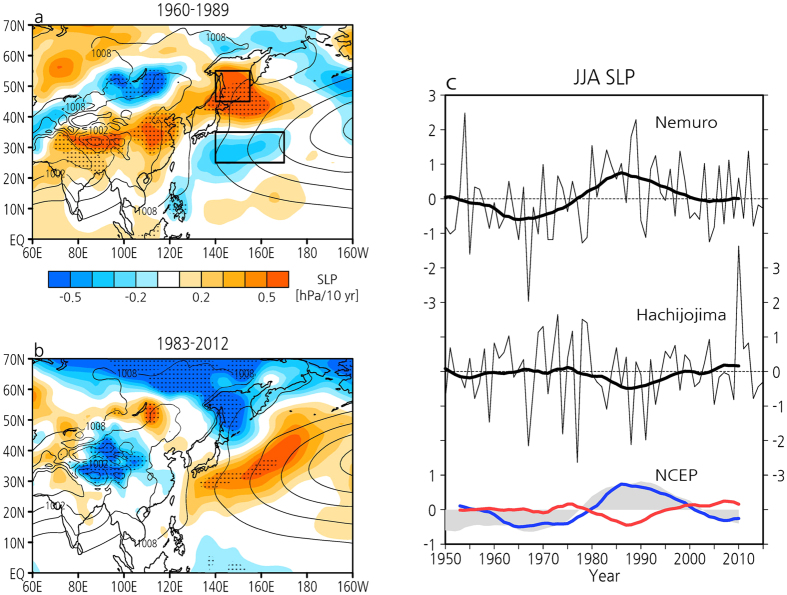
Observed SLP trends and its time series in summer. The spatial pattern of linear trends (decade^−1^) in SLP (hPa) during summertime (JJA) for the period (**a**) 1960–1989 and (**b**) 1983–2012. Black dots indicate statistical significance at the 95% level. Thin black contours indicate mean SLP (3 hPa contour interval). (**c**) Time series of JJA normalized SLP at Nemuro, Japan (43°N, 145°E), Hachijo Island, Japan (33°N, 139.8°E), and averaged over the Sea of Okhotsk (45°–55°N, 140°–155°E) (blue line) and the western Pacific (25°–35°N, 140°–170°E) (red line) surrounded by black rectangles in (**a**). Shading indicates the JJA PDO index for the period 1950–2015. Thick lines and the PDO index indicate 11-year running means. All plots and maps are generated by GrADS version 2.1.0 (http://cola.gmu.edu/grads/).

We mainly use the most reliable reanalysis data (JRA55^16^) over the past five decades across the 1970s, when assimilation of satellite data increased substantially. Over East Asia, especially northern China, sea level pressure (SLP) intensified from 1960 through the 1980s, but then weakened slightly from the 1980s onwards (Fig. 1a and b). The Coupled Model Intercomparison Program phase 5 (CMIP5^17^) models capture well the intensified anticyclonic circulation caused by the weakening of the EASM and the recent recovery of EASM circulation^18^. Over the NW Pacific, on the other hand, the WPSH weakened along the climatological ridge, while SLP over the Sea of Okhotsk, namely the Okhotsk high^19^, intensified before the 1980s. However, since the 1980s, changes in the two highs exhibit completely opposite polarity, as demonstrated in our previous study^7^. Consequently, the WPSH and the Okhotsk high have also shown remarkably contrasting changes in the periods before and after the 1980s. To confirm the reanalysis data, we show the time series of SLP at Hachijo Island in the NW Pacific and Nemuro in northern Japan, which is adjacent to the Sea of Okhotsk (Fig. 1c). Nemuro has an upward trend before the mid-1980s, while at Hachijojima it slightly has a downward trend from the 1960s through the mid-1980s. However, since the mid-1980s, Nemuro has a downward trend and Hachijojima has an upward trend, which is consistent with the reanalysis data. The SLP variations over the Sea of Okhotsk and the NW Pacific along the climatological ridge surrounded by black rectangles in Fig. 1a also show clearly opposite trends with low-frequency variability in the periods before and after the 1980s based on the NCEP-NCAR reanalysis^20^ (Fig. 1c). Interestingly, the low-frequency variability since 1960 corresponds well with the summer PDO index (shaded in Fig. 1c). These station and reanalysis data suggest that the WPSH and the Okhotsk high may be associated with the PDO.

**Figure 2:**
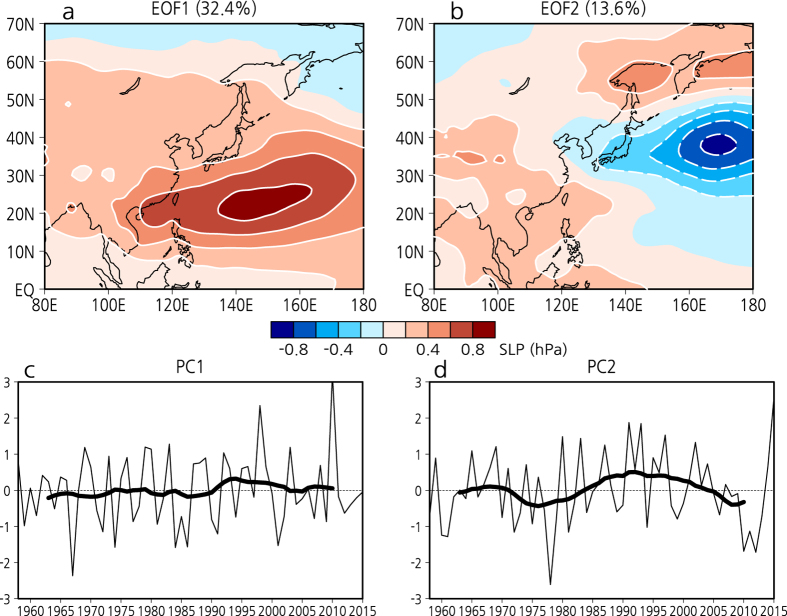
First and second EOF modes of JJA SLP. (**a**) First and (**b**) second EOF modes of JJA SLP (hPa) and corresponding PCs ((**c**) and (**d**)) for the period 1958–2015. Thick black lines indicate 11-year running means. All plots and maps are generated by GrADS version 2.1.0 (http://cola.gmu.edu/grads/).

Our hypothesis that the NW Pacific SLP variations are related to the PDO is supported by an empirical orthogonal function (EOF) analysis. Figure 2 shows the first and second EOF modes of JJA SLP over East Asia and the NW Pacific for the period 1958–2015, which explain 32.4% and 13.6% of the total variance, respectively. SLP EOF-1 dominates the East Asian and the NW Pacific summer climate, which is characteristic of the WPSH. In EOF-2 the SLP anomalies decrease in the NW Pacific and increase around the Sea of Okhotsk, resulting in the contrasting north–south SLP anomalies, although there are other weak positive anomalies in East and Southeast Asia. The EOF-2 patterns resemble the contrasting north–south SLP changes in the periods before and after the 1980s (Fig. 1a and b). The first principal component (PC) shows no pronounced trend, which means that the SLP EOF-1, with its large variance, represents interannual variability. In contrast, PC-2 shows a well-defined low-frequency variability that corresponds closely to the PDO index and station data (Fig. 1c), indicating that the SLP EOF-2 represents decadal variability.

**Figure 3:**
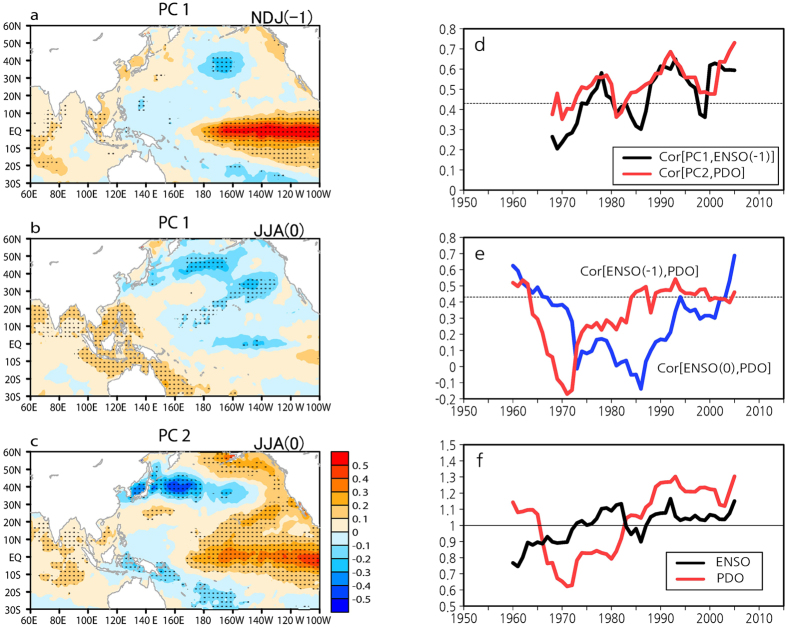
Regressions of SST onto SLP EOFs and interdecadal changes in ENSO and the PDO. (**a**) The preceding (-1) winter (NDJ) and (**b**) the following (0) summer SST anomalies (°C) regressed onto PC-1. Black dots indicate statistical significance at the 95% level. (**c**) As in (**b**), but for PC-2. (d) 21-year running correlations between PC-1 and the preceding ENSO index (black line) and between PC-2 and PDO index (red line). Dotted line denotes the 95% significant level. (**e**) As in (**d**), but for correlation with PDO index: the preceding (red line) and the following (blue line) ENSO indices for the period 1950–2015. (**f**) Normalized 21-year running standard deviations of ENSO (black line) and PDO (red line) indices. All plots and maps are generated by GrADS version 2.1.0 (http://cola.gmu.edu/grads/).

To further examine both EOF modes, we show the SST anomalies regressed onto the PCs (Fig. 3a–c). The enhanced WPSH in EOF-1 is caused by the preceding winter El Niño and concurs with the tropical Indian Ocean SST warming following El Niño, which is characteristic of the ENSO-induced WPSH^11^,^12^,^13^. In addition, dipolar SST anomalies in the Indo-Pacific warm pool can help to sustain the WPSH intensification through a positive feedback^3^. Consequently, the SLP EOF-1 can be viewed as an air–sea interaction mode in the tropical oceans. On the other hand, EOF-2 strongly represents a positive phase of the PDO, demonstrating that the contrasting north–south SLP anomalies are associated with the PDO. The correlation of PC-1 with the preceding ENSO index displays a rapid rise in the 1970s, and then dips in the 1980s (Fig. 3d), which is consistent with the results of previous study^21^. The correlation of PC-2 with the summer PDO index generally exceeds the 95% significant level throughout the analysis period. The dips in correlation around 1970 and 1980 may be related to a decrease in PDO variability, as shown later. Both the correlations tend to have an upward trend throughout the analysis period. These results also support the fact that the first and second EOF modes accurately capture ENSO and PDO variations, respectively.

However, the changes in the PC correlations with the preceding ENSO and the PDO resemble each other, possibly giving rise to suspicion about whether the PDO is independent of ENSO^22^. Now we consider the relationship between ENSO and PDO. Figure 3e shows 21-year running correlations of the preceding (–1) and the following (0) ENSO indices with the summer PDO index for the period 1950–2015. Both ENSO(–1) and ENSO(0) correlations with PDO are high in the 1960s, but rapidly fall around 1970. Subsequently, the ENSO(–1) correlation rises in the mid-1980s and generally maintains significance at the 95% level, while the ENSO(0) correlation barely rises in the 1990s and has recently exceeded the ENSO(–1) correlation, indicating that the ENSO–PDO relationship differs in the decaying and developing ENSO years. ENSO activity intensified from the 1970s through the 2000s, while the PDO was inactive during the 1970s but intensified from the 1980s through the 2000s (Fig. 3f). These results suggest that the relationship between ENSO(–1) and the PDO has also strengthened, as a result of the strong activity of both ENSO and the PDO since the 1980s. For instance, our EOF-1 differs from the EOF-1 of previous study^23^ (for the period 1979–2009 based on the same reanalysis data), which appears to capture the Pacific–Japan teleconnection (PJ) pattern^24^. This difference from our EOF-1 might be the result of the strengthening nature of the relationship between ENSO(–1) and the PDO since the 1980s. The PJ-like pattern appears in our EOF-3 together with equatorial central Pacific cooling (Supplementary Fig. S1). In addition to time dependence, EOF analysis is also domain dependence. In particular, the PJ-like pattern appears in different EOF modes (e.g., EOF-1^23^, EOF-2^3^, and EOF-3 in this study), strongly depending on EOF domain.

**Figure 4:**
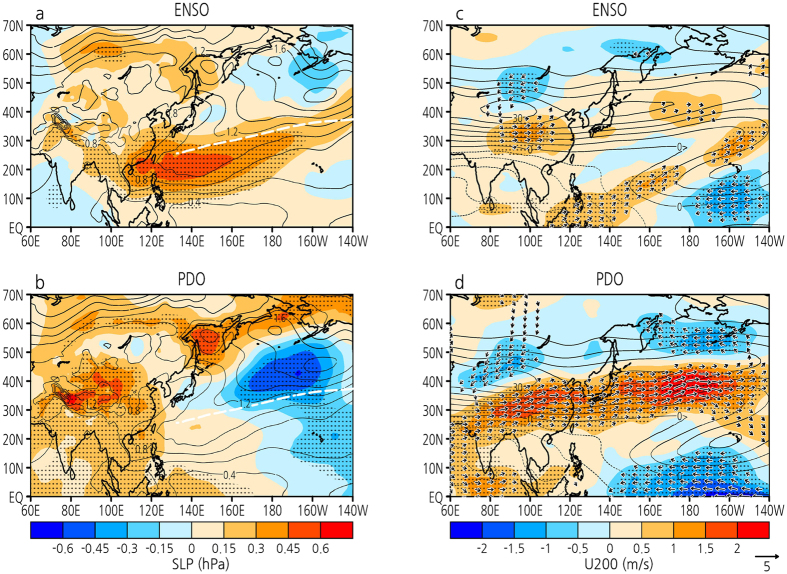
Regressions of SLP and upper tropospheric wind onto ENSO and the PDO. Summer detrended SLP anomalies (hPa) regressed onto (**a**) the preceding ENSO and (**b**) PDO indices for the period 1958–2015. Black dots indicate statistical significance at the 95% level. Thin black contours indicate standard deviation (0.2 hPa contour interval) and white dashed line indicates climatological ridge in the North Pacific subtropical high. (**c**) and (**d**): As in (**a**) and (**b**), but for zonal wind (shadings; m s^−1^) and wind (vectors) anomalies at 200 hPa. Thin black contours indicate mean zonal wind (5 m s^−1^ contour interval). All plots and maps are generated by GrADS version 2.1.0 (http://cola.gmu.edu/grads/).

The preceding ENSO and the PDO have been closely connected since the 1980s, but the mechanisms by which they affect WPSH variations are different in each case. Figure 4a and b shows the SLP anomalies regressed onto the preceding ENSO and PDO indices. The ENSO-induced WPSH intensification is consistent with the SLP EOF-1. The PDO leads to the contrasting north–south SLP anomalies between the Sea of Okhotsk and the NW Pacific, and corresponds closely to the SLP EOF-2. The contrasting north–south SLP anomalies are fundamentally different from those in winter and spring when the Aleutian low develops (Supplementary Fig. S2a). The ENSO-induced WPSH is active along the south side of the climatological ridge in the North Pacific subtropical high, while the PDO-induced negative SLP anomaly over the NW Pacific is active along the north side of the climatological ridge. The north–south responses of the SLP anomalies across the ridge indicate that both ENSO and the PDO are responsible for the WPSH variability. Indeed, the recent intensification of the WPSH is located along the north side of the climatological ridge (Fig. 1b), suggesting that a negative phase of the PDO has affected the intensification of the WPSH.

The mechanisms associated with the PDO that leads to the WPSH variation can be explained as the subtropical jet response to the KOE SST variability. Figure 4c and d shows the 200-hPa zonal wind and wind anomalies regressed onto the preceding ENSO and PDO indices. Over the NW Pacific, ENSO is not a major driver of the variability of the subtropical jet, but the PDO accelerates the westerlies to the south side of the climatological jet and decelerates the westerlies to its north side. Consequently, the positive (negative) phase of the PDO enhances the southward (northward) shift of the subtropical jet through a response to the decreased (increased) KOE SST (i.e., by an SST frontal shift)^25^, which in turn intensifies the cyclonic (anticyclonic) circulation over the NW Pacific and the anticyclonic (cyclonic) circulation over the Sea of Okhotsk^8^. The contrasting north–south circulation anomalies caused by the KOE SST frontal shift appear even if ENSO signal is removed^26^. In winter and spring when the Aleutian low develops, the PDO accelerates the westerlies in the climatological jet core, forming an air–sea interaction with the Aleutian low, whereas in summer and autumn the PDO enhances the meridional shift of the subtropical jet (Supplementary Fig. S2). This result suggests that the jet response to the KOE SST forcing is pronounced in the warm season.

The developing ENSO that is related to the equatorial Pacific sector in the PDO cannot also be ignored, because the ENSO(0) correlation with the PDO has been significantly high since the 2000s, exceeding the ENSO(–1) correlation (Fig. 3e). The influence of the preceding ENSO is largely restricted to the tropical troposphere^12^, whereas the developing ENSO leads to the Pacific and North America (PNA)-like teleconnection (Supplementary Fig. S3), indicating that ENSO forcing is completely different in the preceding and developing ENSO. The PNA-like teleconnection, which is different from the conventional PNA, is induced by the tropical Pacific SST and has recently become enhanced^27^. The recently high ENSO(0) correlation suggests that the developing ENSO has also been linked to the PDO. Indeed, the PNA-like teleconnection has a similar phase to the PDO over the North Pacific (Fig. S3), contributing to the meridional shift of the subtropical jet. However, the developing ENSO-induced PNA-like teleconnection has little influence on the contrasting north–south SLP pattern, which supports the result that the KOE SST is a major driver of the SLP variability.

To confirm our observational analysis, we conducted EOF analysis for the period 1950–2005 using the CMIP5 models (Supplementary Fig. S4). Although the preceding ENSO forcing to the EASM region is too weak in the CMIP5 models^28^, the observed SLP EOF-1 is partially reproduced. In particular, simulated SLP EOF-2s well capture the contrasting north–south SLP pattern together with the PDO, supporting the observational analysis that the PDO-induced pattern is a robust mode as the second forcing of WPSH variability. Under global warming, the WPSH tends to weaken as a result of weakened meridional temperature gradient^29^, which may be associated with the PDO, especially the meridional shift of the subtropical jet response to the KOE SST frontal shift.

## Discussion

As noted in our previous paper^7^, the location of the WPSH in this study is different from the conventional definition, which is located to the west edge of the WPSH, while we focus on the climatological ridge. The conventional WPSH index well captures ENSO-induced WPSH variability^12^,^30^. However, ENSO is the leading forcing of WPSH variability over interannual timescale, and there is no pronounced trend (Fig. 2). Consequently, the conventional definition of the WPSH represents its interannual variability, while our defined region along the climatological ridge (Fig. 1) can be a good indicator for long-term changes in the WPSH, because the causes that lead to the WPSH variation are different.

The winter PDO is strongly coupled with the Aleutian low, which enhances the PDO as atmospheric forcing. As a result, in summer when the Aleutian low disappears, the PDO’s amplitude is weaker than that in winter^10^. However, as the subtropical jet response to the KOE SST forcing is pronounced in summer, the summer PDO (oceanic forcing) enhances atmospheric variability, such as the WPSH. Thus, the winter PDO can be viewed as an air–sea interaction with the Aleutian low, while the summer PDO can be interpreted as a coupling with the WPSH. On the other hand, the variation of the Aleutian low can also be understood as the atmospheric appearance of PDO^31^ (i.e., the North Pacific oscillation). Similarly, in summer when the WPSH develops to take the place of the Aleutian low, the WPSH variation could be understood as the North Pacific oscillation. However, because the PDO is not a single phenomenon but is instead the result of a combination of different physical processes^10^, the North Pacific oscillation could not also be a single phenomenon. Although long-term changes in the WPSH can be explained as the subtropical jet response to the KOE SST variability, it is possible that another process also contributes to enhance the WPSH variation.

The PDO is linked with decadal changes in the strength of ENSO teleconnection globally^10^,^32^. Over the NW Pacific, the preceding ENSO is not a major driver of the variability of the subtropical jet, but over northern East Asia, it enhances the meridional shift of the subtropical jet (Fig. 4c). The preceding ENSO accelerates the westerlies to the south of the climatological jet core and decelerates the westerlies to its north, thereby intensifying the cyclonic circulation, which also appears in its long-term changes^6^,^15^. This can be explained by a mechanism in which the zonally asymmetric tropical SST distribution plays a role in producing a subtropical jet core to the north of the tropical warm pool^33^. The preceding ENSO induces SST warming in the tropical Indian Ocean and the South China Sea (e.g., Fig. 3b). The ENSO-induced tropical Indian Ocean SST anomalies appear to enhance the meridional shift of the subtropical jet over northern East Asia. It is also showed that only the tropical SST forcing accounts for the trends in the meridional jet shift over northern East Asia using an atmospheric general circulation model^15^. As a result, tropospheric cooling and the EASM weakening trend^6^ can be interpreted as a result of the ENSO-induced tropical Indian Ocean warming through the meridional jet shift. However, the PDO also enhances the meridional jet shift over northern East Asia (Fig. 4d) with tropical Indian Ocean warming (Fig. 3c). The tropical Indian Ocean warming is a common variation to the preceding ENSO and the PDO. It is possible that the strong connection between the preceding ENSO and the PDO since the 1980s (Fig. 3e) is induced by the recently enhanced tropical Indian Ocean SST anomaly related to the preceding ENSO^21^. The tropical Indian Ocean might hold the key to understanding the ENSO–PDO relationship.

## Methods

### Data and model

We used the Japanese 55-year reanalysis (JRA55)^16^ and also the National Centers for Environmental Prediction/National center for Atmospheric Research (NCEP-NCAR) reanalysis^20^ for comparison. To confirm the consistency of the reanalysis data, SLP data from observational stations were obtained from the Japan Meteorological Agency (available online at http://www.data.jma.go.jp/obd/stats/etrn/index.php). SST data were obtained from the Hadley Centre Global Sea Ice and Sea Surface Temperature (HadISST)^34^. We refer to NDJ (November–January) SST averaged over the eastern equatorial Pacific (Nino-3.4: 5°S–5°N, 120°–170°W) as the ENSO index^12^. The PDO index was obtained from the University of Washington (available online at http://research.jisao.washington.edu/pdo/PDO.latest). We also used the historical simulations (Supplementary Table S1) from the CMIP5 models^17^ to confirm observational results. All of the model data were horizontally interpolated onto the 2.5° × 2.5° grid points before analysis. The period 1950–2005 was used, as this is the period covered by the data available for the historical simulation.

### Analyses

To determine the major modes of SLP variations, we performed an empirical orthogonal function (EOF) analysis of JJA (June–August) SLP over the NW Pacific and East Asia (0°–70°N, 80°E–180°). Before the EOF analysis, SLP anomaly data were weighted by the cosine of latitude to ensure that equal areas were afforded equal weight in the analysis. To examine interdecadal variations, we performed a 21-yr running correlation, before which a 9-yr running mean was applied to remove decadal and longer variations^21^.

## Additional Information

**How to cite this article**: Matsumura, S. and Horinouchi, T. Pacific Ocean decadal forcing of long-term changes in the western Pacific subtropical high. *Sci. Rep.* **6**, 37765; doi: 10.1038/srep37765 (2016).

**Publisher's note:** Springer Nature remains neutral with regard to jurisdictional claims in published maps and institutional affiliations.

## Supplementary Material

Supplementary Information
