# Supplementary material for: Pacific Ocean decadal forcing of long-term changes in the western Pacific subtropical high
Source: Sci Rep. 2016 Nov 30;6:37765. doi: 10.1038/srep37765 (PMC5129182; doi:10.1038/srep37765)
Supplement: Supplementary Information [file srep37765-s1.pdf]

Supplementary information for

Pacific Ocean decadal forcing of long-term changes in the western Pacific subtropical high

Shinji Matsumura[1*] and Takeshi Horinouchi[1]

Contents of this file

Figures S1 to S4

Table S1

[1] Faculty of Environmental Earth Science, Hokkaido University, Kita 10 Nishi 5, Sapporo 060-0810, Japan,

* Corresponding author: Shinji Matsumura (e-mail: matsusnj@ees.hokudai.ac.jp)

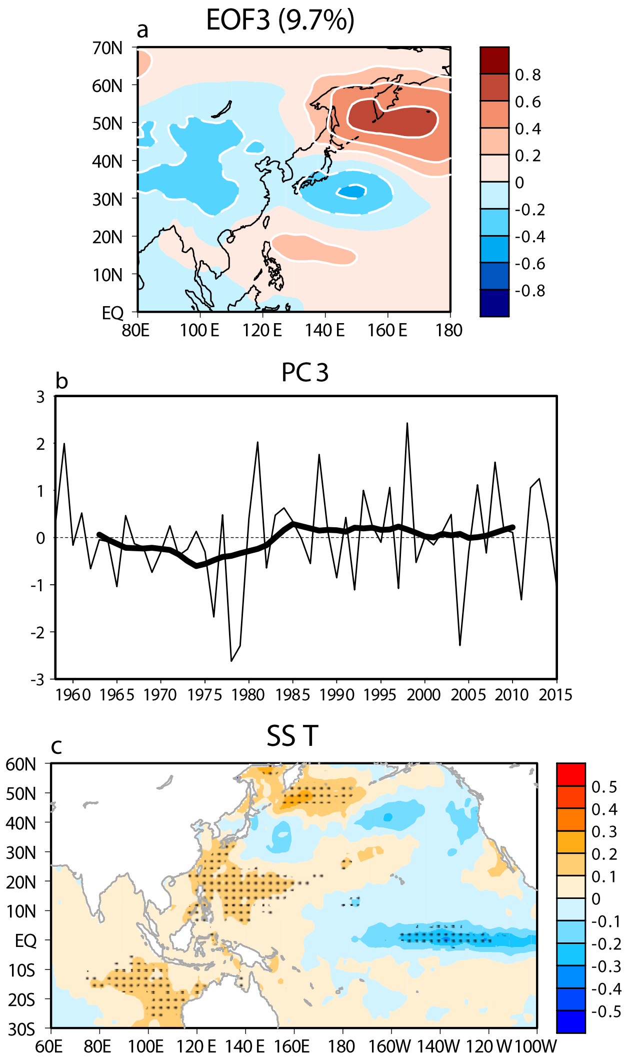

Figure S1 | Third EOF mode of JJA SLP. (a) Third EOF mode of SLP (hPa) and (b) corresponding PC. Thick black lines indicate 11-year running means. (c) Summer SST anomalies (°C) regressed onto PC-3. Black dots indicate statistical significance at the 95% level. All plots and maps are generated by GrADS version 2.1.0 (http://cola.gmu.edu/grads/).

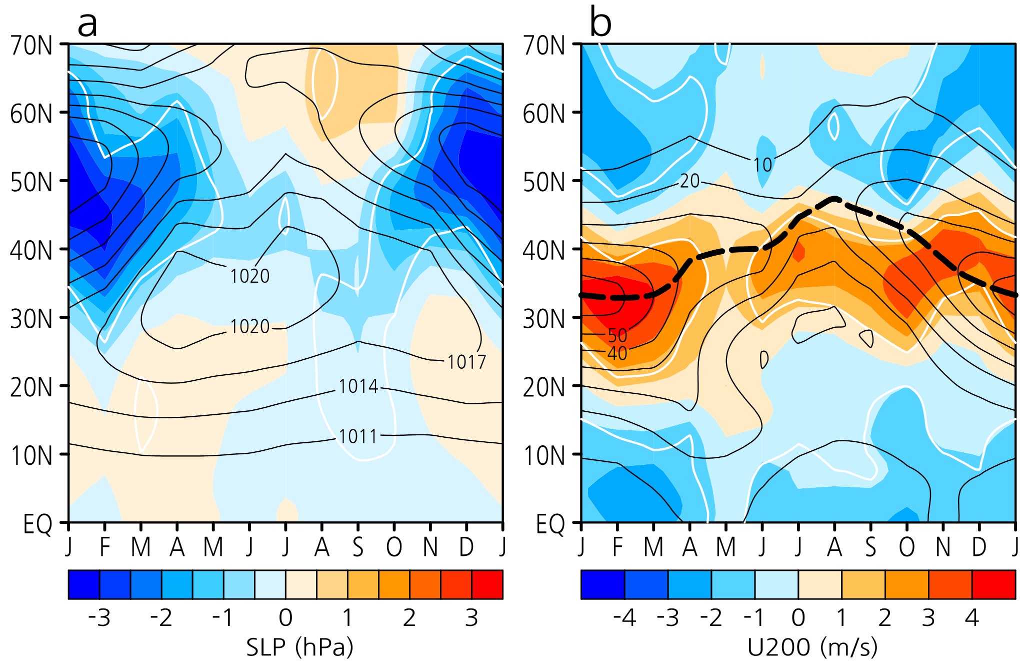

Figure S2 | Seasonal evolution of regressions of SLP and upper tropospheric wind onto the PDO. Time-latitude section of detrended regression onto the PDO index as a function of calendar month: (a) SLP over the North Pacific (160°E–140°W) and (b) 200-hPa zonal wind (m s^{-1}) over 140°E–160°W for the period 1958–2015. White contours indicate statistical significance at the 95% level. Thin black contours indicate mean (a) SLP (3 hPa contour interval) and (b) 200-hPa zonal wind (10 m s^{-1} contour interval), and thick dashed line indicates the subtropical jet axis. All plots and maps are generated by GrADS version 2.1.0 (http://cola.gmu.edu/grads/).

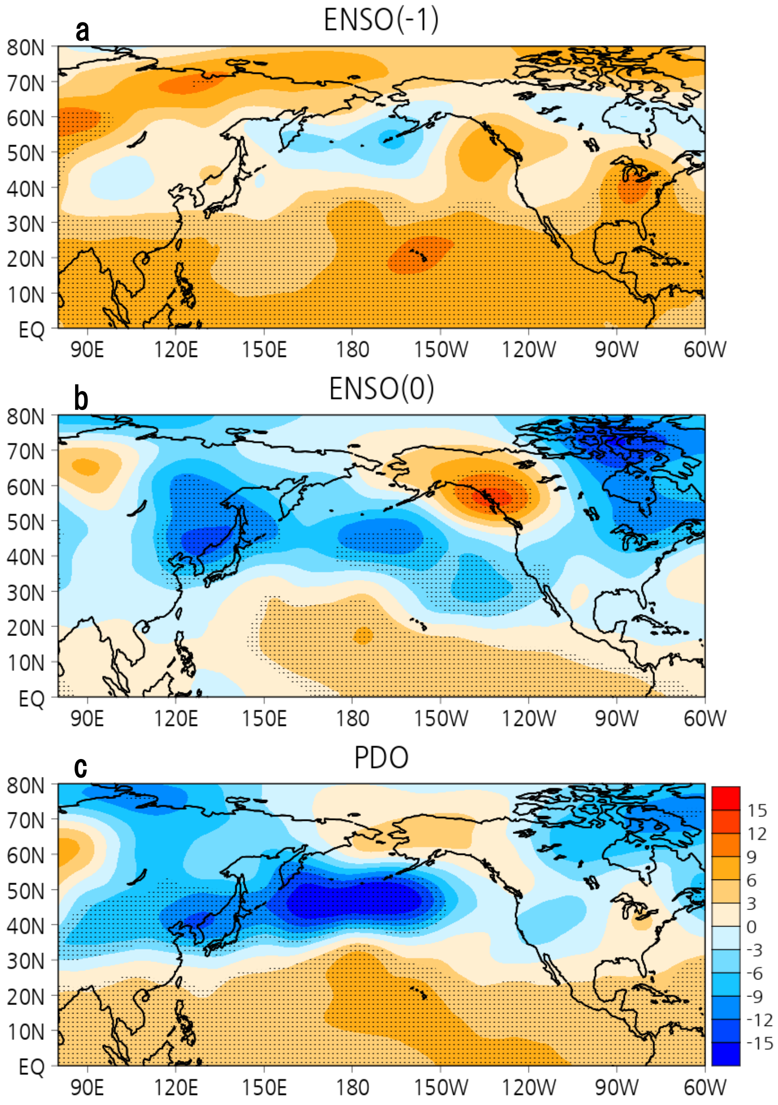

Figure S3 | Regressions of upper tropospheric geopotential height onto ENSO and the PDO. Summer detrended 300-hPa geopotential height anomalies (m) regressed onto (a) the ENSO(-1), (b) ENSO(0), and (c) PDO indices for the period 1958–2015. Black dots indicate statistical significance at the 95% level. All plots and maps are generated by GrADS version 2.1.0 (http://cola.gmu.edu/grads/).

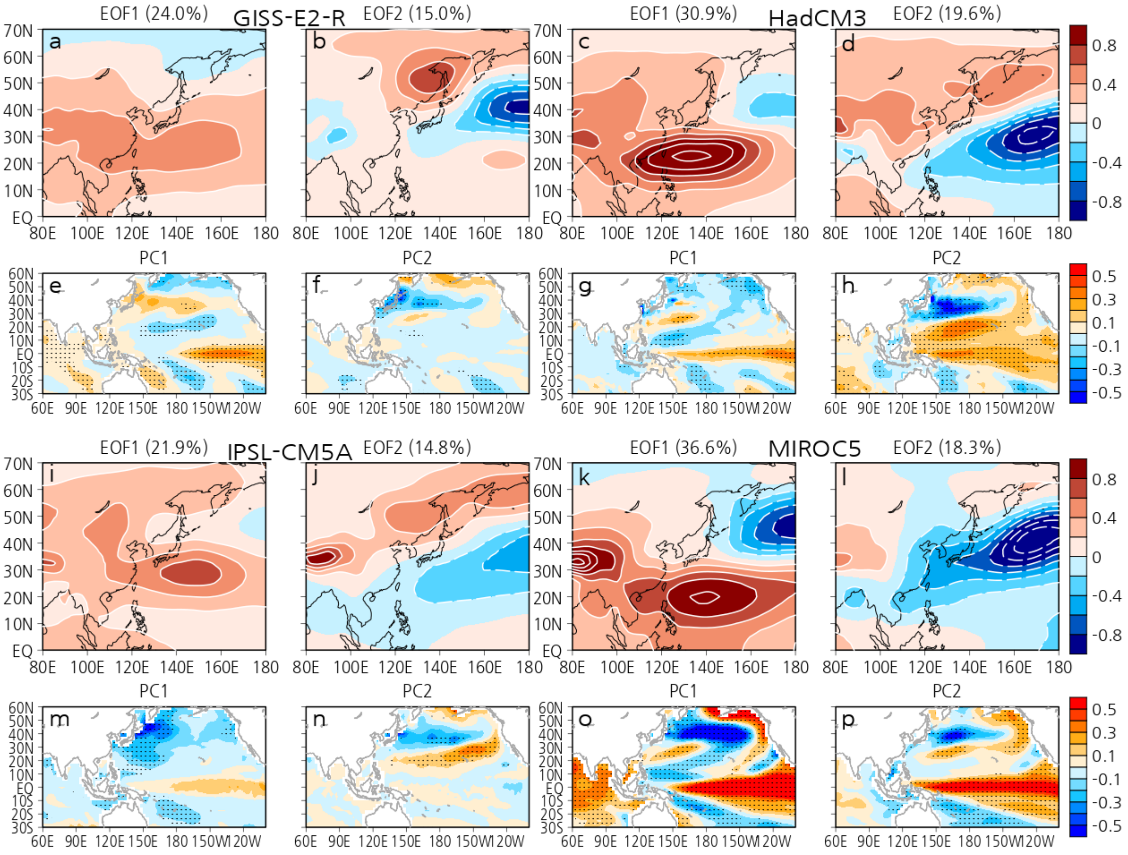

Figure S4 | First and second EOF modes of JJA SLP based on the CMIP5 models. First and second EOF modes of SLP (hPa) for the period 1950–2005 in (a) and (b) GISS-E2-R, (c) and (d) HadCM3, (i) and (j) IPSL-CM5A-LR, and (k) and (l) MIROC5. (e) and (f), (g) and (h), (m) and (n), and (o) and (p) Summer SST anomalies (°C) regressed onto corresponding PCs. Black dots indicate statistical significance at the 95% level. All plots and maps are generated by GrADS version 2.1.0 (http://cola.gmu.edu/grads/).

Table S1 CMIP5 Models used in this study.

No.	Model	Institution	Resolution (lon × lat)
1	GISS-E2-R	NASA-GISS	144 × 90
2	HadCM3	MOHC	96 × 73
3	IPSL-CM5A-LR	IPSL	96 × 96
4	MIROC5	MIROC	256 × 128
